# Supplementary material for: Mutations on the surface of HDAC1 reveal molecular determinants of specific complex assembly and their requirement for gene regulation
Source: Nucleic Acids Res. 2025 Sep 17;53(17):gkaf918. doi: 10.1093/nar/gkaf918 (PMC12448893; doi:10.1093/nar/gkaf918)
Supplement: gkaf918_Supplemental_Files [file gkaf918_supplemental_files.zip › Supplementary Figures - Fig S1-S7.pdf]

Supplementary Fig S1

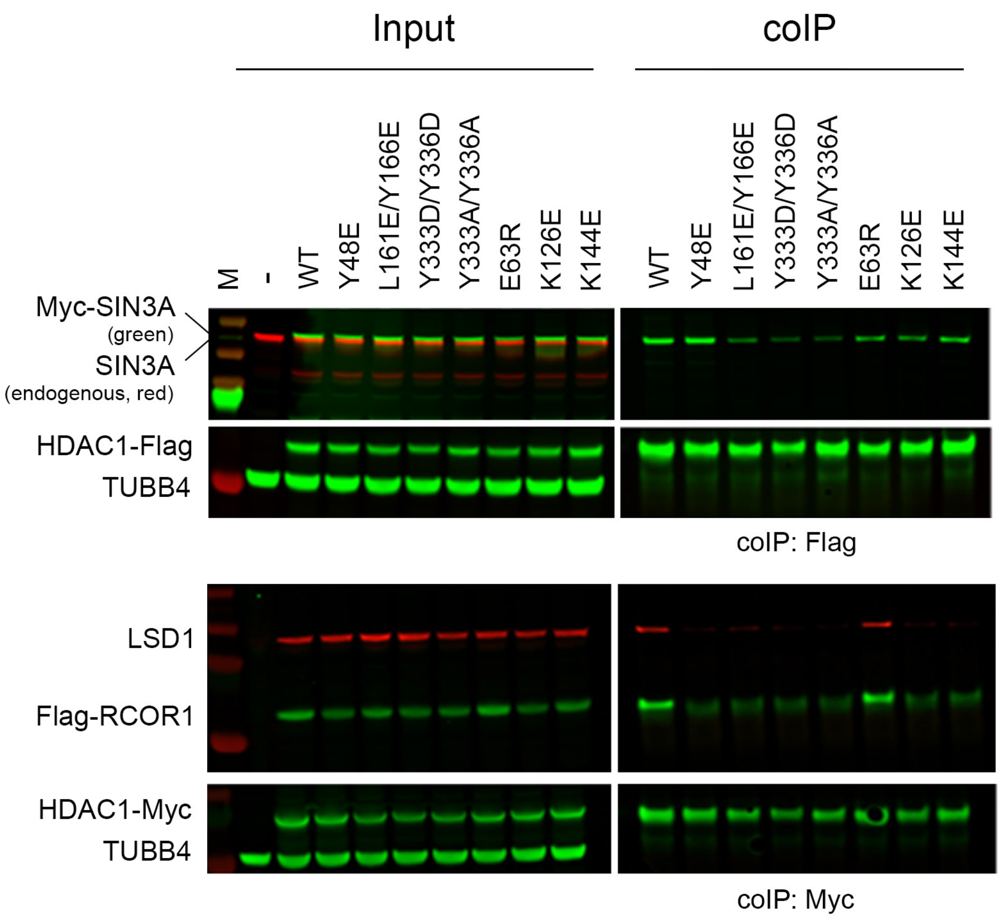

Fig S1 - **HDAC1 surface mutations show differential binding to corepressor partners.** Wildtype or mutant HDAC1 with a Flag-tag or Myc-tag (as indicated) were immunoprecpitated from HEK293T cells. Western blot shows input or binding (coIP) of the indicated proteins. TUBB4 was used as a loading control.

Supplementary Fig S2

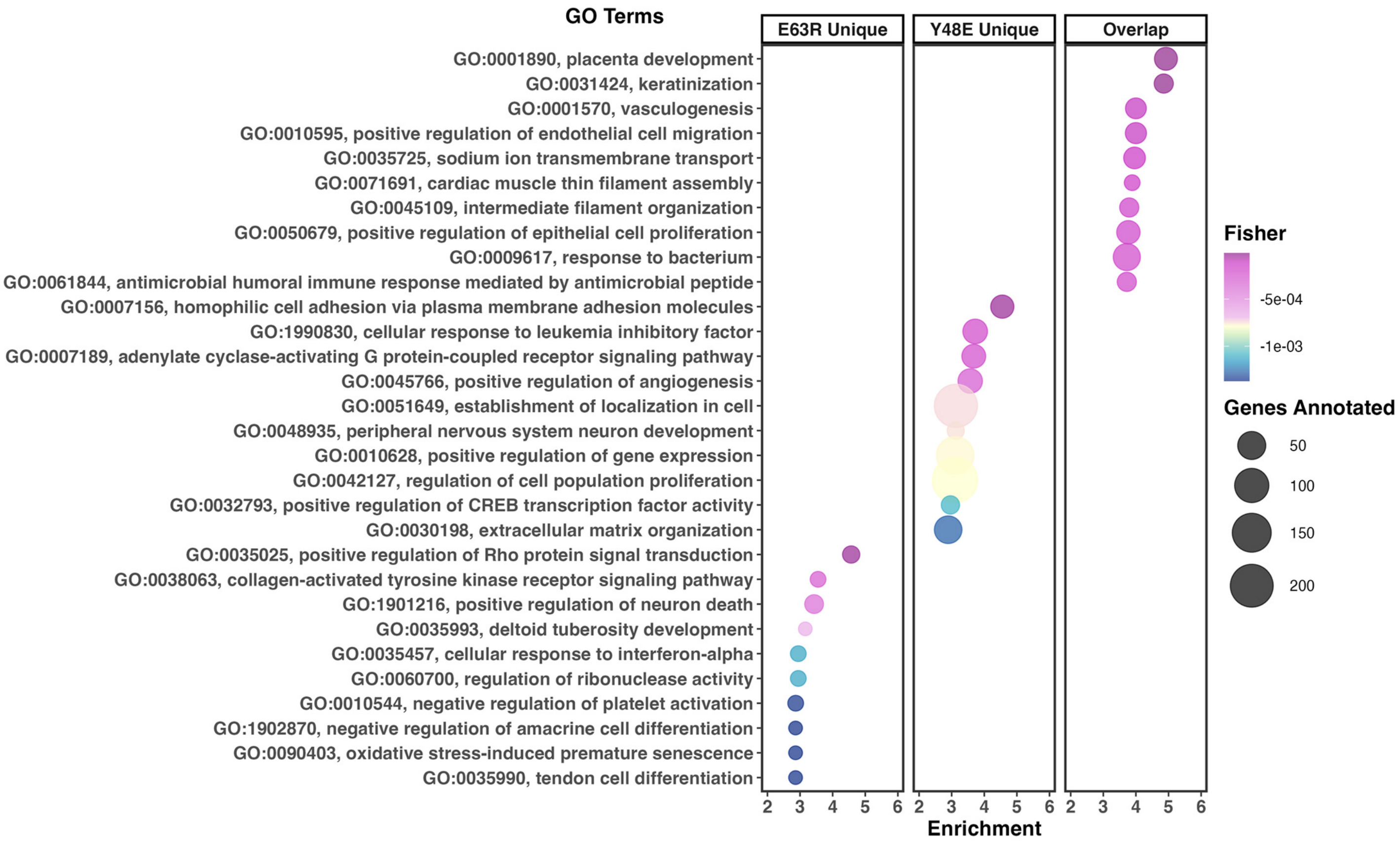

Fig S2 - **Gene ontology (GO) analysis of differentially expressed genes (DEGs).** The 10 most enriched biological process GO terms associated with each individual mutant (*E63R unique* and *Y48E unique*) or shared (*overlap*) are shown (padj <0.01, $\log_2$ fold change > -1) following 24 hours of HDAC1-dTAG degradation.

Supplementary Fig S3

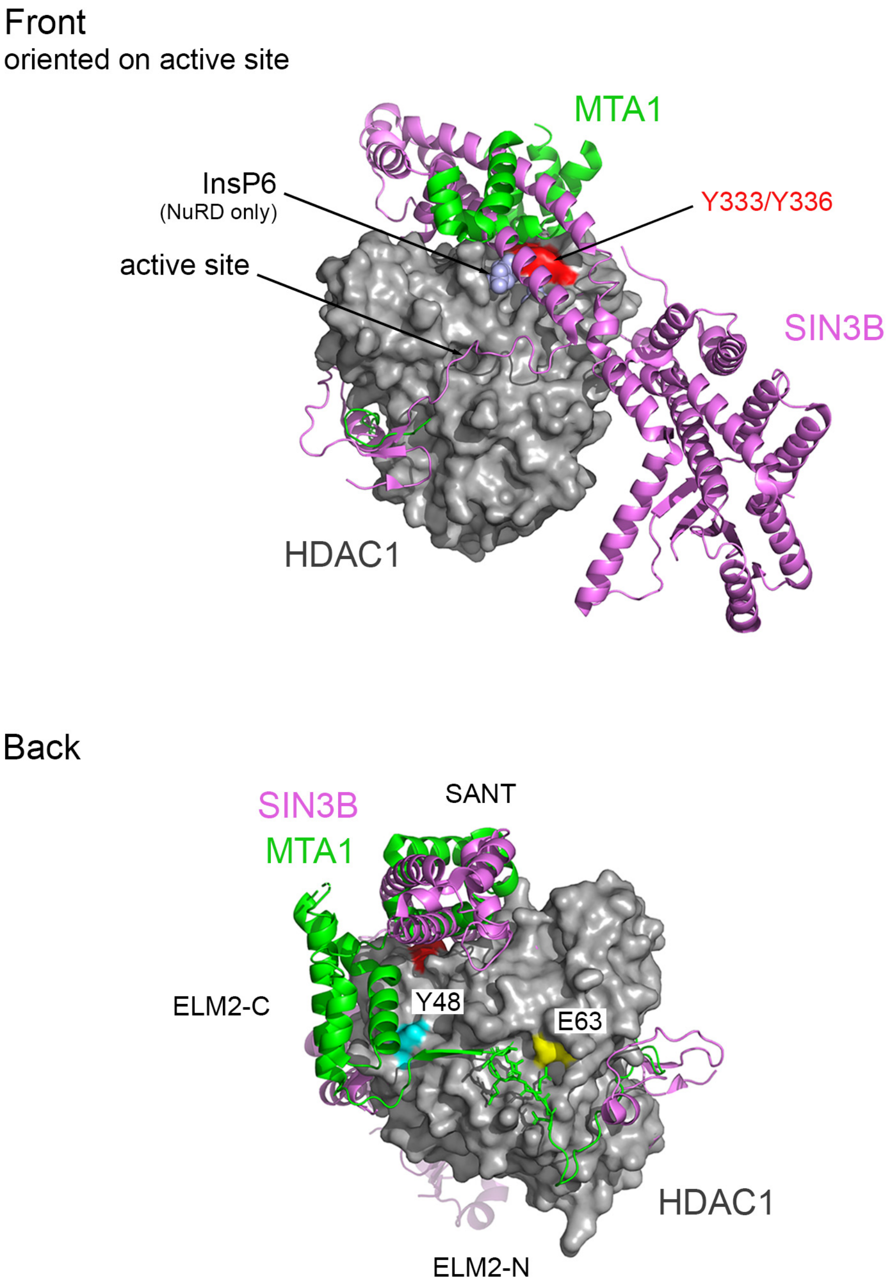

Fig S3 - **HDAC1 binding proteins have distinct modes of interaction.** Binary structures of MTA/HDAC1 (5ICN) and SIN3B/HDAC2 (8BPA) are superimposed to demonstrate different binding modalities. Residues required for interaction with binding partners are labelled. The front of HDAC1 (based on the position of the active site, as indicated) is shown above, with the back below. InsP6 is present only in the NuRD complex, but has been included as a reference point.

Supplementary Fig S4

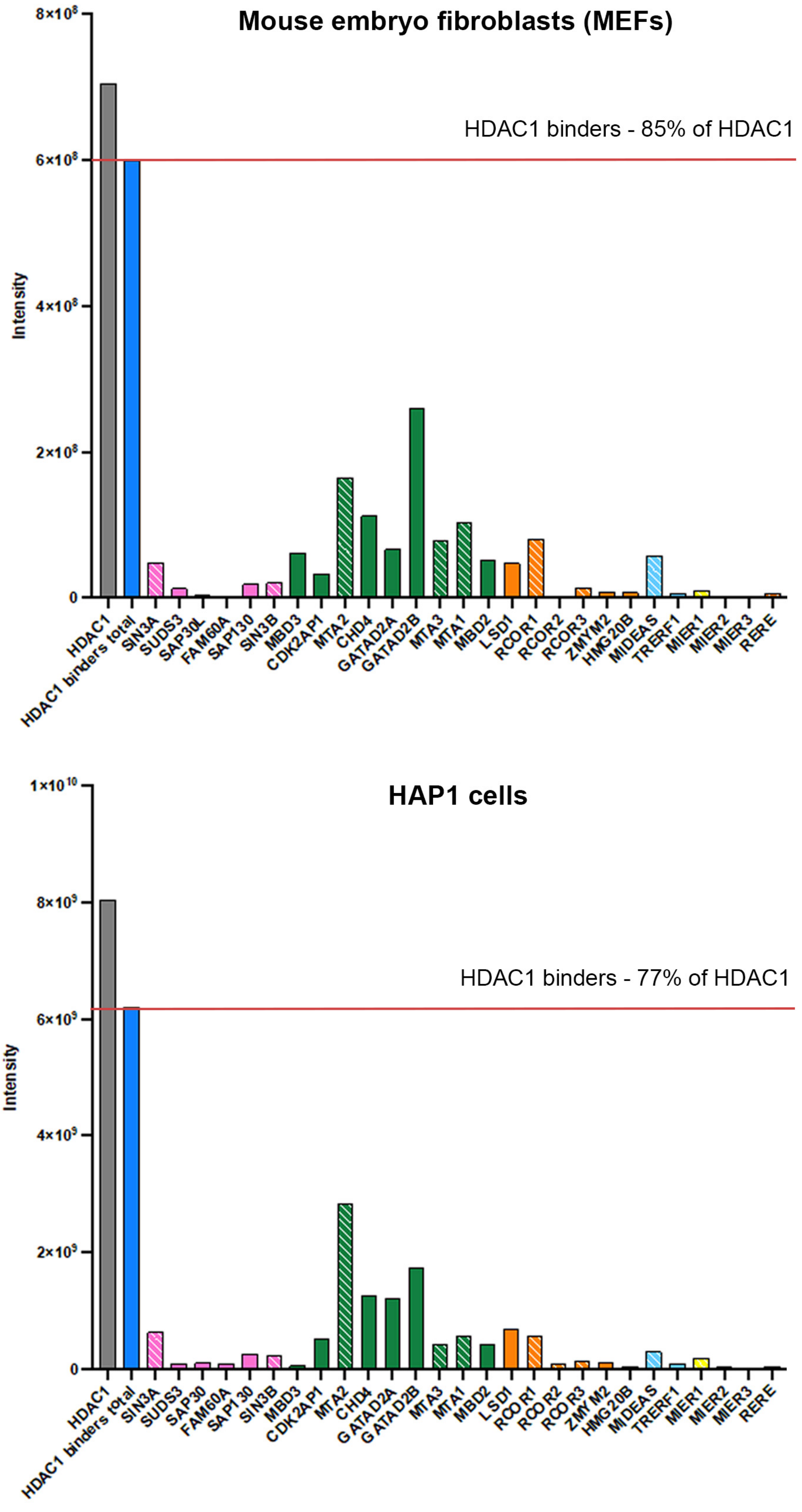

Fig S4 - **HDAC1 co-immunoprecipitation / mass-spectrometry data taken from Vcelkova et al., 2023 (PMID:37878419)**. Intensity values for individual components of HDAC1/2 containing complexes are shown from MEFs and HAP1 cells. Direct HDAC1 binding proteins are indicated by hashed lines.

Supplementary Fig S5

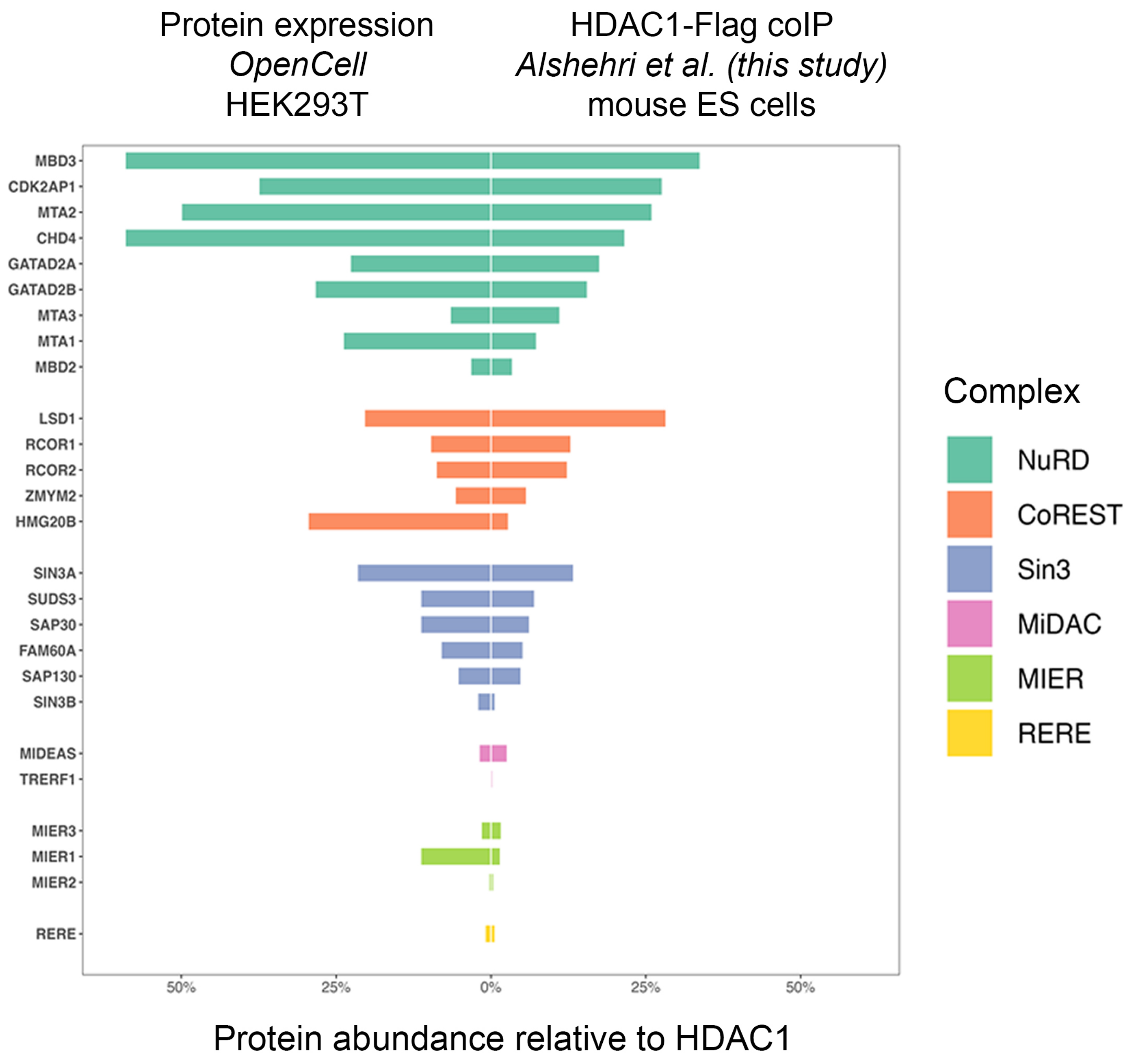

Fig S5 - **Comparison of HDAC1/2 complex component levels**. Graph shows protein abundance levels relative to HDAC1 based on protein copy number estimates from the OpenCell resource (PMID: 35271311) in HEK293T cells (left side) and the HDAC1-flag experiments from this study. Specific proteins are indicated on the left handside and coloured relative to their specific HDAC1/2 complex incorporation.

Supplementary Fig S6

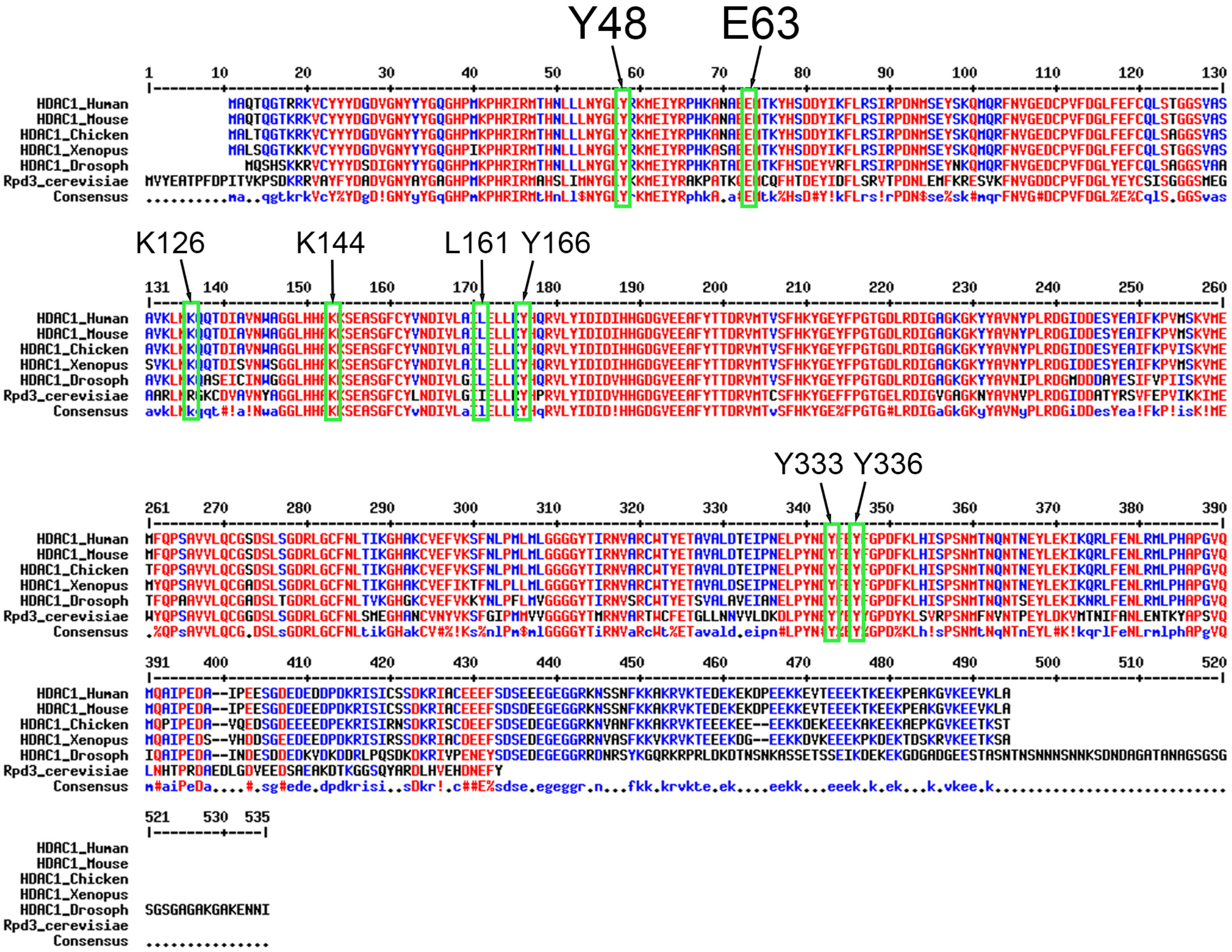

Fig S6 - **Alignment of HDAC1 from different species shows conservation of residues examined in the study**. The protein sequences from the indicated species were aligned using Multalin (PMID: 2849754). Residues examined in the study are hihlighted in green and labelled as shown.

Supplementary Fig S7

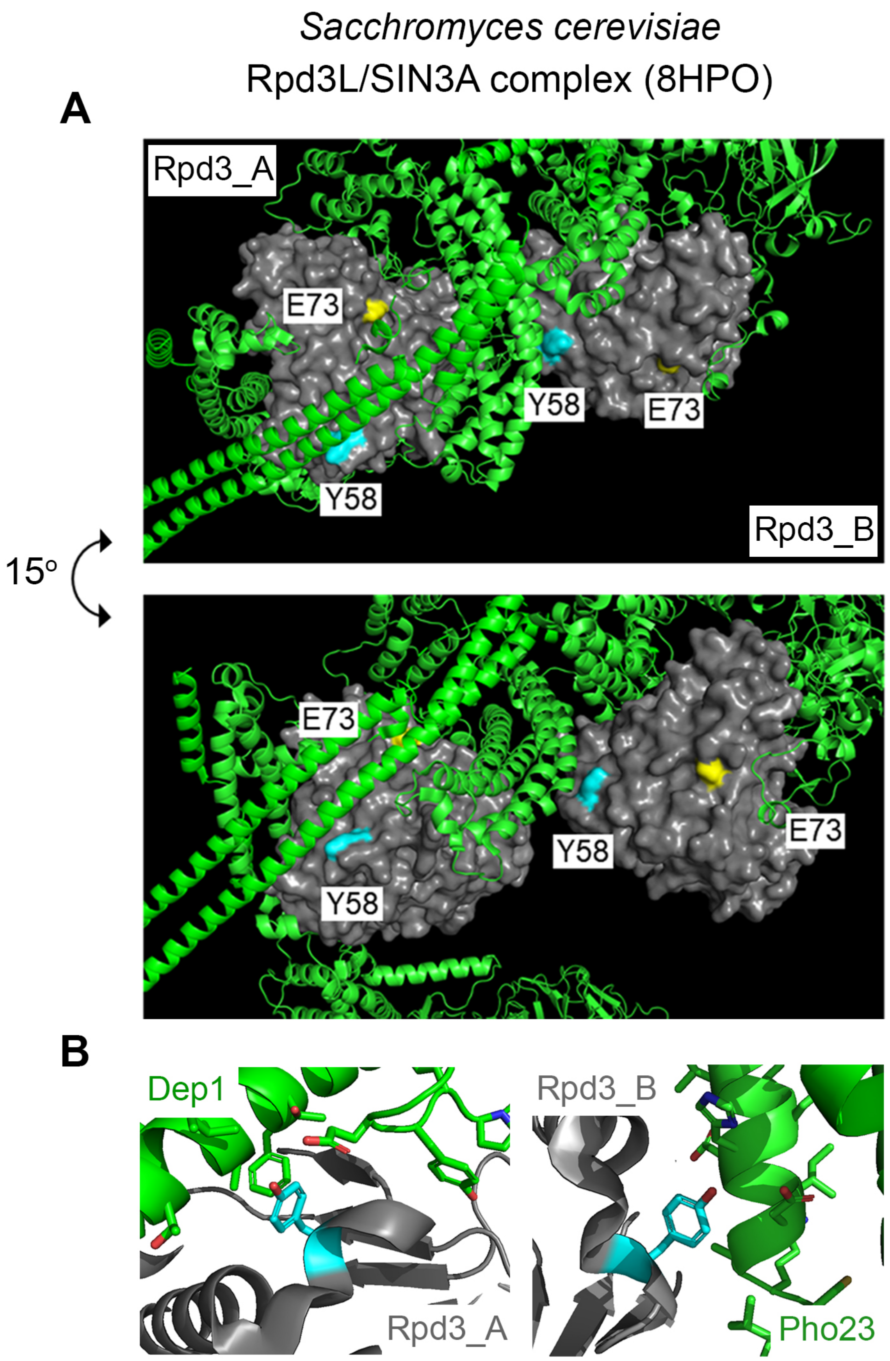

Fig S7 - **Y48 and E63 residues are conserved in the S. cerevisiae Rpd3L complex**. (A) Rpd3L complex (8HPO) was used to model interactions of equivalent HDAC1 residues Y48 (Y58) and E63 (E73). The surface of both copies of Rpd3 (A and B) are shown in grey with the positions of Y58 (cyan) and E73 (yellow) indicated. (B) The position on Y58 is shown in relation to adjacent proteins within the complex, Rpd3_A/Dep1 and Rpd3_B/Pho23. Pymol detected no interactions within 4 angstroms for Y58 in either Rpd3 subunit.
